# A gene module identification algorithm and its applications to identify gene modules and key genes of hepatocellular carcinoma

**DOI:** 10.1038/s41598-021-84837-y

**Published:** 2021-03-09

**Authors:** Yan Zhang, Zhengkui Lin, Xiaofeng Lin, Xue Zhang, Qian Zhao, Yeqing Sun

**Affiliations:** 1 College of Environmental Science and Engineering, Dalian Martime University, Linghai Road, Dalian, 116026 Liaoning China; 2 College of Information Science and Technology, Dalian Maritime University, Linghai Road, Dalian, 116026 Liaoning China

**Keywords:** Cancer, Computational biology and bioinformatics, Biomarkers

## Abstract

To further improve the effect of gene modules identification, combining the Newman algorithm in community detection and K-means algorithm framework, a new method of gene module identification, GCNA-Kpca algorithm, was proposed. The core idea of the algorithm was to build a gene co-expression network (GCN) based on gene expression data firstly; Then the Newman algorithm was used to initially identify gene modules based on the topology of GCN, and the number of clusters and clustering centers were determined; Finally the number of clusters and clustering centers were input into the K-means algorithm framework, and the secondary clustering was performed based on the gene expression profile to obtain the final gene modules. The algorithm took into account the role of modularity in the clustering process, and could find the optimal membership module for each gene through multiple iterations. Experimental results showed that the algorithm proposed in this paper had the best performance in error rate, biological significance and CNN classification indicators (*Precision*, *Recall* and *F-score*). The gene module obtained by GCNA-Kpca was used for the task of key gene identification, and these key genes had the highest prognostic significance. Moreover, GCNA-Kpca algorithm was used to identify 10 key genes in hepatocellular carcinoma (HCC): CDC20, CCNB1, EIF4A3, H2AFX, NOP56, RFC4, NOP58, AURKA, PCNA, and FEN1. According to the validation, it was reasonable to speculate that these 10 key genes could be biomarkers for HCC. And NOP56 and NOP58 are key genes for HCC that we discovered for the first time.

## Introduction

With the development of sequencing technology, a lot of transcriptome data have emerged. Among them, genes have the characteristics of modularized function. To be specific, the expression levels of genes with the same function are often similar, the so-called “co-expression”, which provides a basis for identifying gene modules from gene expression data. At present, the gene module identification methods are mostly based on Gene Co-expression Network Analysis (GCNA). The concept of gene co-expression network (GCN) was first proposed by Butte and Kohane in 1999, and they constructed the first GCN based on the Pearson correlation analysis of gene expression data^1,2^. Recently, the most commonly used algorithm in GCNA is Weighted Gene Co-expression Network Analysis (WGCNA)^3^, which identifies gene modules based on the idea of hierarchical clustering and combines the two tasks of “GCN construction” and “gene module identification” in one process.

Although the WGCNA algorithm has been widely used to identify gene modules, it still has some shortcomings need to be improved. Firstly, WGCNA algorithm is based on network clustering, but it fails to take modularity^4^ into account in module identification process. Modularity is an index proposed by Newman et al. to evaluate the community detection results. And the community detection refers to the clustering of nodes in the network using the topology of the network. A community corresponds to a cluster (gene module). Modularity plays an important role in network clustering and community detection, and clustering results with high modularity are usually more reliable. Secondly, since the WGCNA algorithm is based on hierarchical clustering, once it is determined which branch of the tree that a gene belongs to during the execution of the algorithm, it cannot be undone. Which means the algorithm cannot find the best membership module for each gene with multiple iterations. These above two points might induce the WGCNA algorithm could not obtain the optimal gene modules. To optimize the gene module identification method, we combined community detection and K-means algorithm framework to propose a new gene module identification method. Finally, experiments were conducted to verify the reliability of the proposed algorithm.

In the last decade, the high-throughput platforms were used to generate gene expression profiling in hepatocellular carcinoma (HCC). However, sequencing results are often limited and inconsistent owing to the heterogeneity of samples in independent studies. As such, this study sought to analyze a range of available HCC-related gene expression data sets by proposed algorithm, with the goal of identifying key gene module and genes for HCC treatment and diagnosis.

Above all, we downloaded the gene expression profile of HCC from the Cancer Genome Atlas (TCGA)^5^ and preprocessed it. Next, the algorithm proposed in this paper and seven algorithms were used to identify the gene modules in HCC, respectively. Then we compared the identification effects of the eight algorithms. Then, a key module was selected in the identification result of the algorithm we proposed, and we performed GO enrichment analysis on it. Besides, to identify key genes, key modules identified by K-means, WGCNA and GCNA-Kpca were used to construct protein protein interaction (PPI) network with Search Tool for the Retrieval of Interacting Genes (STRING) database^6^. And the identification effects of three algorithms were compared with two key gene identification algorithms which were most commonly used. Finally, key genes were validated by three methods, Oncomine analysis, GEO data set and ROC curve.

## Materials and methods

### Sources of data

The HCC gene expression profiles used in this study were downloaded from TCGA (https://cancergenome.nih.gov), which were processed using the RNA-sequencing platform, and contained 416 samples, including 367 HCC samples and 49 normal samples. The data preprocessing method mainly included the four steps:

1. The low-expression genes were filtered. That was, the gene whose maximum FPKM value was less than 1 in HCC or normal samples was removed.
2. Outliers from HCC samples were removed by hierarchical clustering with R function hclust() in the stats package (v3.6.1), and samples whose cluster height were significantly higher than most samples were removed (In this study, TCGA-DD-AAEB, TCGA-CC-5259 and TCGA-FV-A4ZP are removed, see Fig. S1).
3. The fold change of each gene’s FPKM value between HCC and normal samples was calculated, and genes with FC ≥ 2 (up-regulated) or FC ≤ 0.5 (down-regulated) were retained. The cutoff values were obtained by combining the need for subsequent analysis and referring to reference^7–9^.
4. T-test was performed on the genes retained in step (3) using the t.test() in stats R package (v3.6.1). The significance of the difference in RPKM values of each gene between HCC and normal samples was tested, and the genes with P-value < 0.05 were retained.

### Construction of GCN

Chang et al. showed that when Pearson correlation analysis was performed on the expression levels of two genes, if the absolute value of the correlation coefficient was greater than a certain threshold and met statistical significance, it could be considered that the two genes have a co-expression interaction^10^. In this paper, Pearson correlation analysis was used to calculate the similarity between the two genes’ expression levels. If the absolute value of the Pearson correlation coefficient (PCC) of the two genes was greater than the given threshold (|PCC|≥ 0.65) and met statistical significance (P-value < 0.05), the two genes were considered to have a co-expression interaction. All co-expression interactions were represented by networks, which was GCN.

### Community detection algorithm

The community detection algorithm is a kind of clustering algorithm, which divides the nodes in the network into several communities (clusters) based on the network topology. The nodes within the community are closely connected, while the nodes between the communities are sparsely connected. In GCNA, a community detection algorithm can be used to divide genes in the network into different communities, and a community is a gene module.

In 2006, Newman proposed a community detection algorithm with the goal of maximizing modularity (called Newman algorithm in this paper)^11,12^. The Newman algorithm takes modularity optimization as the main idea. It can divide genes in the GCN into different communities and realize the identification of gene modules. However, this algorithm is still unable to find the best membership module for each gene through multiple iterations.

### Gene module identification method based on Newman algorithm and K-means algorithm

K-means algorithm is a classical clustering method, and it finds the best membership cluster for each sample point through multiple iterations. But it still has two problems: Firstly, the number of clusters K needs to be determined before the algorithm is executed. Secondly, it is necessary to initialize the clustering center, and the selection of the initial clustering center will have a key influence on the clustering results.

In this study, GCNA-Kpca algorithm was proposed by combining Newman algorithm and traditional K-means algorithm. The core idea is that a GCN is constructed using gene expression data firstly; then Newman algorithm is used to initially identify gene modules based on the topological structure of the GCN, and the number of clusters and clustering centers are determined; finally, the number of clusters and clustering centers are input into the K-means algorithm framework, and secondary clustering is performed based on the gene expression profile to obtain the final gene modules. This algorithm combines the advantages of Newman algorithm and K-means algorithm, and could find the optimal membership module for each gene through multiple iterations, and at the same time makes full use of the topology of GCN and gene expression profiles, so as to identify gene modules more accurately.

However, the traditional K-means algorithm could not achieve good results directly for the identification of gene modules, so we improved the algorithm on two aspects in this study. One is to change the definition of distance. The distance in the K-means algorithm is always defined between a sample point (gene) and a clustering center. The traditional K-means algorithm uses Euclidean distance, which is obviously not suitable for clustering genes. We learned from the method used in the construction of GCN and used the PCC to define the distance. The specific formula is as follows:

1 $$D(g,C) = 1 - \left| {cor(g,C)} \right|,$$

where, $g$ represents a gene, $C$ represents a cluster center, and the calculated result of function $cor()$ is the PCC of the two variables.

The second is to change the strategy of determining clustering center. Before the K-means algorithm is executed, the initial clustering center must be determined; after the K-means algorithm has completed a division of genes, the clustering center must be determined again. To better explain the method of determining clustering center in this paper, the concept of module eigengene (ME) is introduced: In GCNA, a vector ME is often used to represent the expression profiles of all genes in a gene module (cluster). Generally, Principal Component Analysis (PCA) is performed on the expression of all genes in a gene module, in which the first principal component is ME of the module. A study have shown that the stronger the correlation between gene g and the ME of module i, the more likely it is that gene g belongs to module i^13^. Based on this principle, we aimed to find the best membership module for each gene through multiple iterations. Therefore, the MEs of gene modules in the preliminary clustering result of Newman algorithm were used as initial clustering centers of K-means algorithm in this study. The strategy for updating a clustering center was to perform PCA on all genes contained in a cluster, and made the first principal component as the new clustering center.

The process of the GCNA-Kpca algorithm is as follows:

- *Step 1* Let P_n×m_ be the expression matrix of n genes in m samples.
- *Step 2* Pearson correlation analysis is performed for all row vectors in P_n×m_ in pairs to construct a GCN G.
- *Step 3* Use Newman algorithm to recursively split G, and community structure is obtained.
- *Step 4* The number of communities K and ME of each gene module were obtained.
- *Step 5* Initialize the number of clusters as K, and initialize the clustering centers as K MEs.
- *Step 6* Use formula (1) to calculate the distance from each gene to each clustering center.
- *Step 7* Cluster each gene to the nearest clustering center.
- *Step 8* Perform PCA on all genes contained in a cluster, and make the first principal component as a new clustering center.
- *Step 9* Check whether the termination condition is met. If the termination condition is met, the algorithm ends; otherwise, go to Step 6.

### Evaluation indicators for gene module identification

In order to prove the superiority of the GCNA-Kpca algorithm, clustering algorithms based on different principles were used for comparative experiments, including seven algorithms: K-means, K-means++, K-medoids, Gaussian Mixture Model (GMM), Spectral Clustering, Fuzzy c-means (FCM) and WGCNA.

We evaluated the identification effect from the following aspects. One is the error rate of clustering. As we all know, when Pearson correlation analysis is performed between a gene and ME of its corresponding module, the absolute value of the PCC is called the module membership (MM) of this gene^13^. In an ideal situation, genes in the same module should be highly correlated. That is, if there is a gene $g \in$ module i, then for $\forall j \ne i$, there is

2 $$MM_{g} \ge \left| {cor(g,ME_{j} )} \right|.$$

Among them,$MM{}_{g}$ is the MM of gene g, and $ME_{j}$ is the ME of module j. If a gene doesn’t satisfy formula (2), the membership of the gene in its module is low. That is, the gene is wrongly divided into this module. Therefore, the error rate was defined as the ratio of the number of genes that didn’t satisfy the formula (2) to the total number of genes.

The second is the biological significance of the module. Biological process (BP) in the results of Gene Ontology (GO) enrichment analysis can help understand the biological functions that a gene module involves in, and Fisher’s precise test can characterize the significance and reliability of these biological functions. Based on this, we defined the calculation formula of biological significance (Sig_i_) of the i^th^ gene module as follows:

3 $$Sig_{i} = \sum\limits_{j = 1}^{n} { - \log_{10} } (P\;{\text{value}}_{j} ),$$

where, n represents the number of GO terms (BP) in the i^th^ gene module, and $P\;{\text{value}}_{j}$ represents the significance P-value value of Fisher’s exact test corresponding to the j^th^ GO Term in this module. Therefore, the biological significance (Sig) of the results of an algorithm is shown in Formula (4):

4 $$Sig = \sum\limits_{i = 1}^{m} {Sig_{i} } /m,$$

where, m represents the total number of gene modules identified by this algorithm.

After obtaining the labels from clustering, we built supervised classification models using Convolutional Neural Networks (CNN) to further evaluate the reliability of the clustering results. For the clustering results obtained by each algorithm, we constructed a model using the 70% TCGA samples (training set) and predicted the labels in 30% samples (test set), and the evaluation indicators included *Precision*, *Recall* and *F*-score.

### Application of gene modules

In this paper, an important downstream task of gene module identification, the identification of key genes, was selected to further prove the good effect of GCNA-Kpca algorithm in gene module identification, and also to demonstrate the application of this algorithm in bioinformatics analysis.

We selected the key modules (the module with the highest biological significance) in the results of the K-means, WGCNA and GCNA-Kpca, and input genes in the three key modules into the STRING database (https://string-db.org/) respectively to build PPI networks. Then we defined the 10 genes with the highest PageRank algorithm^14^ score in each network as the key genes identified by this algorithm.

### Evaluation indicators for key gene identification

To compare the value of key genes obtained by different algorithms, survival analysis was used to evaluate the reliability of a gene. Generally, if the Logrank P-value of a gene is less than 0.05, it can be considered that the expression level of the gene is significantly correlated with overall survival (OS), and the smaller the P-value, the stronger the correlation. Therefore, the prognostic significance (Sig_SA) of all key genes obtained by an algorithm is defined as shown in Formula (5):

5 $$Sig\_SA = \sum\limits_{i = 1}^{n} { - \log_{10} } (P\;{\text{value}}_{i} ),$$

where, n represents the number of key genes (in this paper n = 10); $P\;{\text{value}}_{i}$ represents the Logrank P-value of the i^th^ gene.

### Verification of key genes

Three methods were used to further verify the role of key genes identified by GCNA-Kpca algorithm: Firstly, the mRNA expression of key genes was explored in common cancer using Oncomine^15^ (https://www.oncomine.org). The parameters were set as follows: threshold (P-value) = 0.05, THRESHOLD (FOLD CHANGE) = 1.5. Then, we downloaded a test data set, GSE138485, from the gene expression omnibus (GEO) (https://www.ncbi.nlm.nih.gov/geo), and this data set included 64 paired normal and HCC samples (Table S1). The t-test was used to verify the differential expression of the key genes in GSE138485. Ultimately, ROC curve and AUC were used to detect the ability of key genes to distinguish tumors from normal tissues.

## Results

### Preprocessing of gene expression data

**Figure 1:**
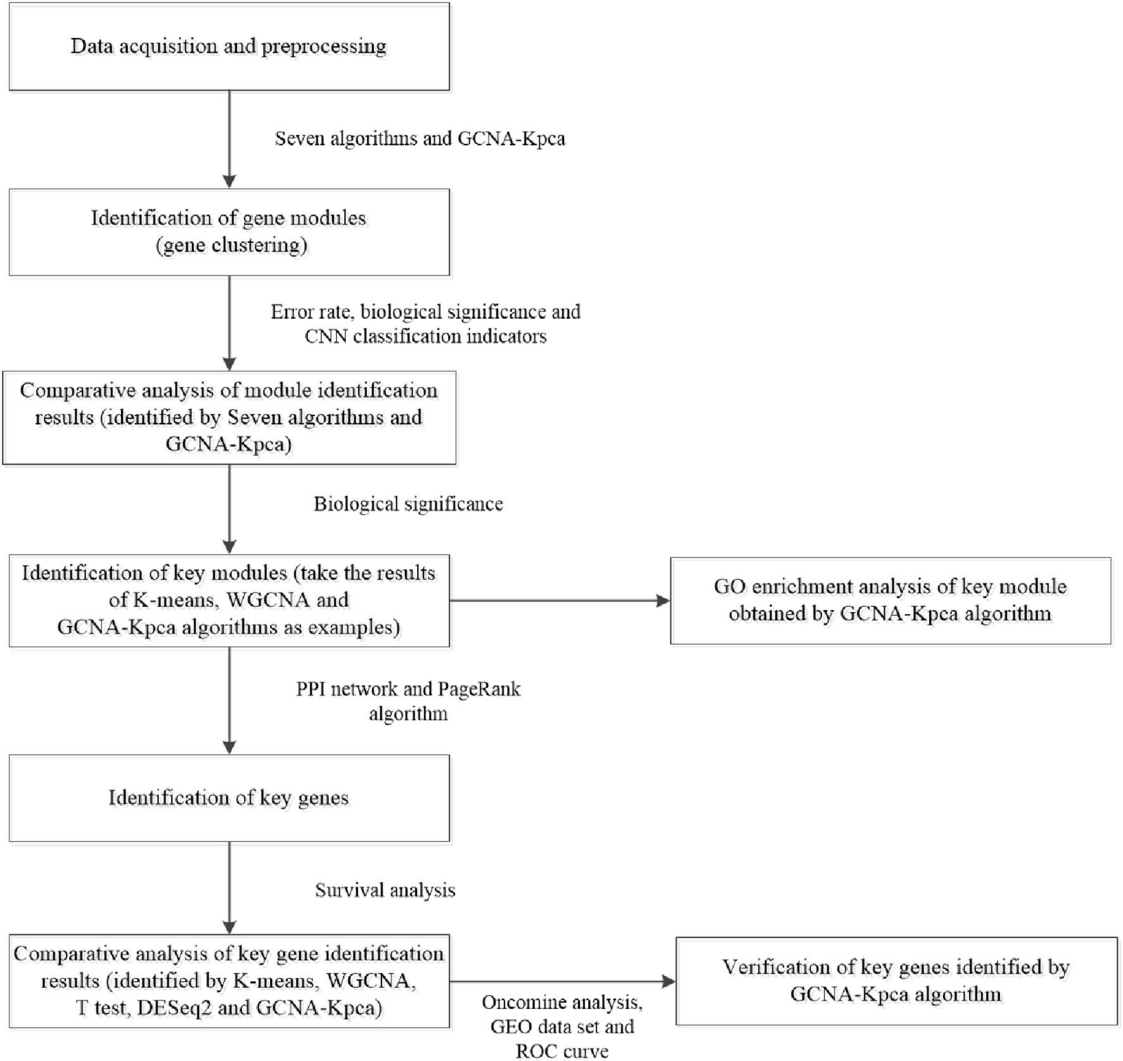
Flow-chart of data analysis in this paper. This figure was drawn with Microsoft Visio 2010.

**Figure 2:**
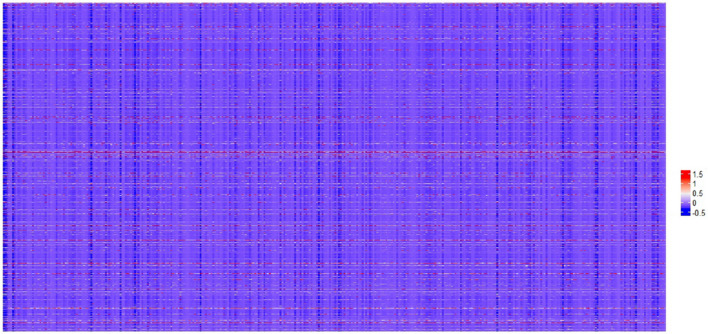
Gene expression profiles of HCC samples. The results obtained by normalizing the RPKM values in 364 HCC samples, each of which contained 4601 genes. A row corresponds to a gene, and a column corresponds to a sample. This figure was drawn with R software^16^.

A workflow of this study is shown in Fig. 1. We preprocessed the gene expression data of HCC firstly, and the gene expression matrix P4601 × 364 was obtained for further analysis (Fig. 2^16^), which contained 4601 genes and 364 samples, all of which were HCC samples.

### Identification of gene modules and comparative analysis of results

Seven algorithms (K-means, K-means++, K-medoids, GMM, Spectral Clustering, FCM, WGCNA) and the GCNA-Kpca algorithm were used to analyze the preprocessed data to identify gene modules. Then, the error rate of the identification results of the eight algorithms was calculated (Table 1). It can be seen that the GCNA-Kpca algorithm has the lowest error rate (0.06). Moreover, the error rate of community detection results using only Newman algorithm is 0.25, indicating that the effectiveness of the GCNA-Kpca algorithm has been greatly improved compared with the Newman algorithm.

**Table 1:** Comparison of error rates among the eight algorithms (K-means, K-means++, K-medoids, GMM, Spectral Clustering, FCM, WGCNA and GCNA-Kpca).

Algorithm	Error rate
K-medoids	0.58
K-means	0.53
Spectral clustering	0.42
GMM	0.39
FCM	0.37
K-means++	0.31
WGCNA	0.29
GCNA-Kpca	0.06

**Figure 3:**
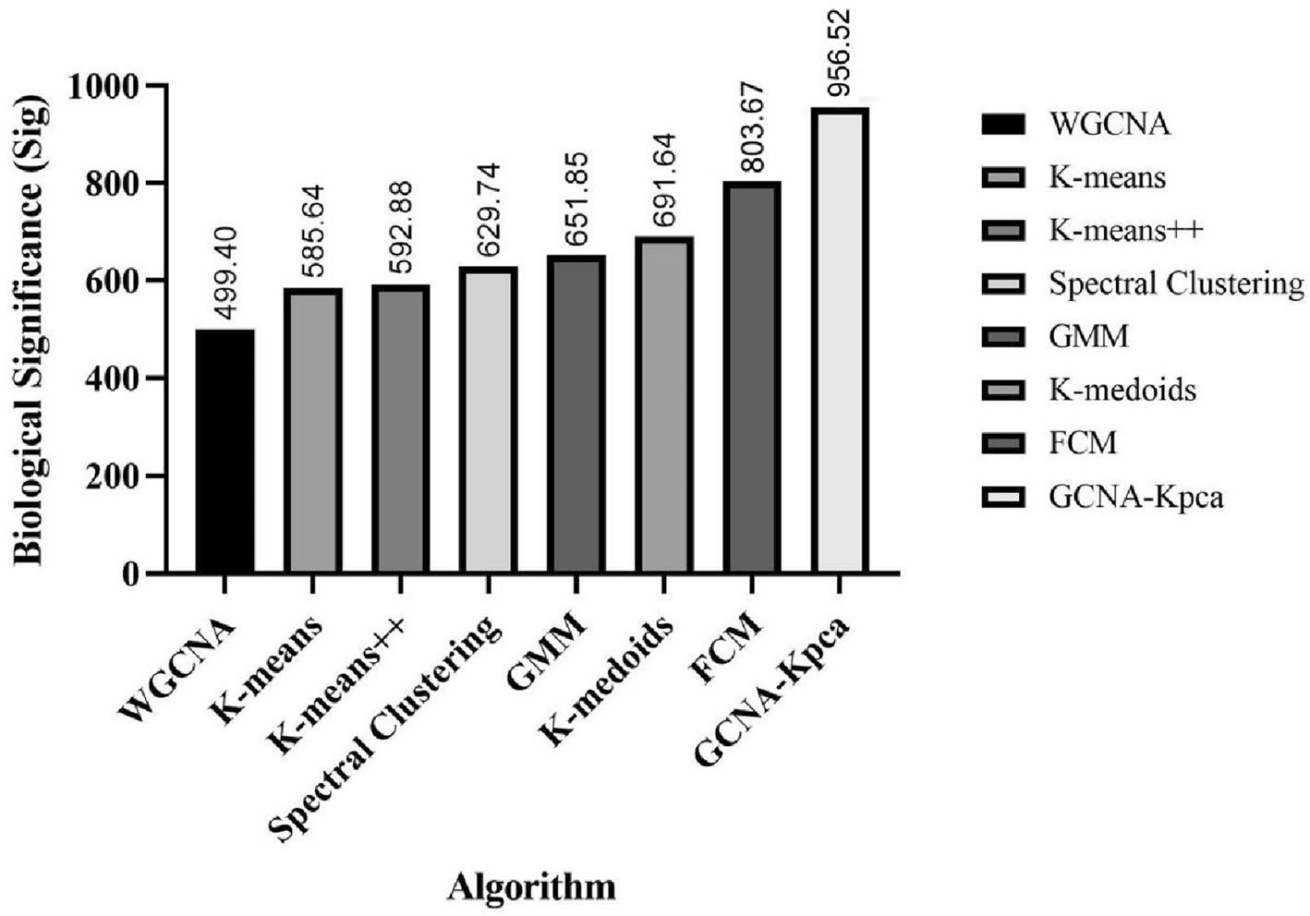
The biological significance of gene modules identified by K-means, K-means++, K-medoids, GMM, Spectral Clustering, FCM, WGCNA and GCNA-Kpca. This figure was drawn with GraphPad Prism 9.

Furthermore, the biological significance of the gene modules identified by the eight algorithms was calculated according to formulas (3) and (4) (Fig. 3). It can be seen that the results obtained by GCNA-Kpca algorithm have the highest biological significance (Sig = 956.52).

Finally, we used CNN to evaluate the clustering results (Table 2). Obviously, our algorithm, GCNA-Kpca, performs the best. It has the highest *Precision* (0.8410), *Recall* (0.7670), and *F*-score (0.7895)*.*

**Table 2:** The classification results of CNN.

Clustering algorithm	Precision	Recall	F-score
K-means++	0.407837302	0.412309368	0.409291088
K-means	0.420653138	0.420298143	0.413029082
GMM	0.458085362	0.479953484	0.467910418
Spectral clustering	0.443782761	0.510982469	0.471149819
K-medoids	0.520787355	0.521708201	0.517168854
FCM	0.720416663	0.688164921	0.690147127
WGCNA	0.78614125	0.729279985	0.741099025
GCNA-Kpca	0.84104302	0.766970131	0.789498886

### Identification and GO enrichment analysis of key module obtained by GCNA-Kpca algorithm

**Figure 4:**
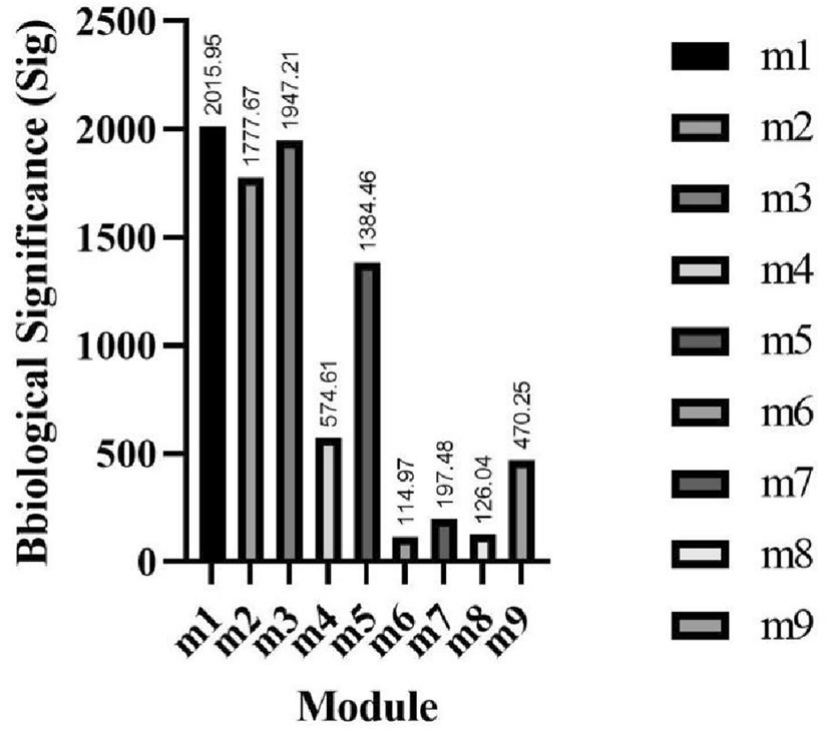
The biological significance of nine gene modules identified by GCNA-Kpca algorithm. This figure was drawn with GraphPad Prism 9.

The biological significance of the nine gene modules identified by GCNA-Kpca algorithm was calculated respectively (Fig. 4). Module m1 had the highest biological significance, so m1 was defined as the key gene module identified by GCNA-Kpca algorithm. Further, GO enrichment analysis was performed on module m1, and the 20 BPs with the smallest P-value were shown in Table 3. The genes in m1 mainly participated in BPs associated with cell cycle process, cytoskeleton organization, and localization.

**Table 3:** The 20 GO Terms (BPs) with the smallest P-value in the key gene module (m1) identified by GCNA-Kpca algorithm.

ID	Description	Count	*P*-value
GO:0044770	Cell cycle phase transition	39	9.53E−17
GO:0044772	mItotic cell cycle phase transition	36	1.99E−15
GO:0034660	ncRNA metabolic process	35	2.35E−15
GO:0051301	Cell division	36	4.07E−15
GO:0016072	rRNA metabolic process	24	9.97E−15
GO:0007051	Spindle organization	21	2.54E−14
GO:0006261	DNA-dependent DNA replication	19	2.73E−14
GO:0010564	Regulation of cell cycle process	39	1.11E−13
GO:0007346	Regulation of mitotic cell cycle	37	1.14E−13
GO:0006403	RNA localization	22	1.50E−13
GO:0034470	ncRNA processing	27	2.19E−13
GO:0000280	Nuclear division	28	2.40E−13
GO:0006260	DNA replication	23	2.80E−13
GO:0140014	Mitotic nuclear division	23	3.03E−13
GO:0006281	DNA repair	32	4.19E−13
GO:0007052	Mitotic spindle organization	16	4.63E−13
GO:0033044	Regulation of chromosome organization	25	2.31E−12
GO:0048285	Organelle fission	28	3.04E−12
GO:1902850	Microtubule cytoskeleton organization involved in mitosis	16	1.37E−11
GO:0000723	Telomere maintenance	17	1.41E−11

### Identification of key genes

**Figure 5:**
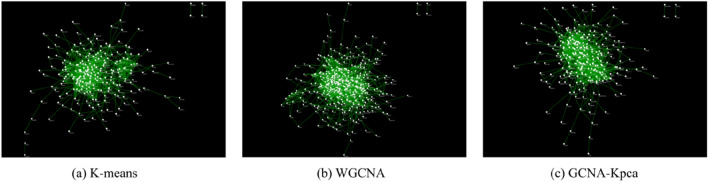
PPI networks of the three key modules. These key gene modules identified by K-means, WGCNA and GCNA-Kpca respectively. And each node in PPI network corresponds to a gene. This figure was drawn with Cytoscape 3.7.1^17^.

We input the key modules identified by the three algorithms (K-means, WGCNA and GCNA-Kpca) into the STRING database to obtain the PPI networks (Fig. 5^17^).

Furthermore, PageRank algorithm was used to identify key genes in three PPI networks. In addition, two of the most commonly used key gene identification algorithms, T test and DESeq2 algorithm^18^, were selected for comparative analysis. These two algorithms directly identify key genes by analyzing gene expression profiles, which is the traditional method for key genes identification. Each algorithm also identified 10 key genes (Table 4).

**Table 4:** Key genes identified by five algorithms (K-means, WGCNA, T test, DESeq2 and GCNA-Kpca).

Algorithm	Key gene
K-means	TOP2A, RFC4, AURKA, ESPL1, MCM2, ZWINT, SMC3, MCM5, RRM2, POLD1
WGCNA	CCNB1, CDC20, TOP2A, RFC4, RBBP7, PCNA, AURKA, FEN1, MCM2, MCM3
T test	PPOX, MSTO1, TOMM40L, DAP3, LRRC14, VPS45, SCAMP3, TMCO1, PRCC, TBCE
DESeq2	ADAMTS13, ANGPTL6, VIPR1, OIT3, ECM1, CSRNP1, CFP, CCL23, CPEB3, CDC37L1
GCNA-Kpca	CDC20, CCNB1, EIF4A3, H2AFX, NOP56, RFC4, NOP58, AURKA, PCNA, FEN1

### Comparative analysis of key gene identification results

**Figure 6:**
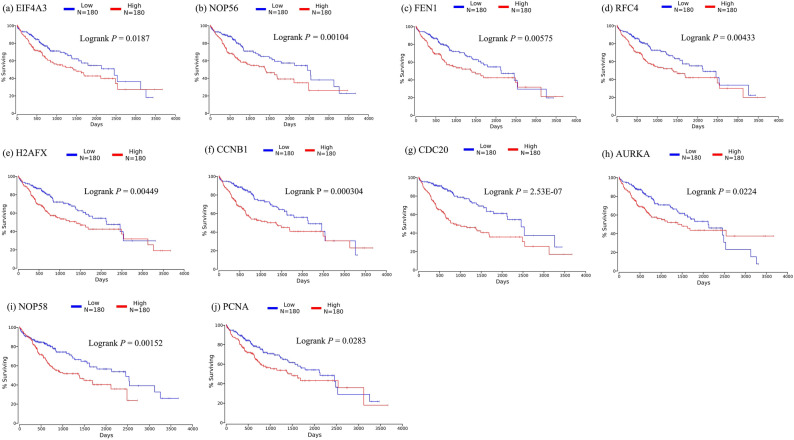
Significant correlation between key genes expression and survival. Survival curves of key genes identified by GCNA-kpca algorithm. X-axis represents survival time and Y-axis represents survival rate. This figure was drawn with OncoLnc^19^ (http://www.oncolnc.org).

The survival analysis of key genes showed that the 10 key genes identified by GCNA-Kpca algorithm were all significantly correlated with OS (Logrank P-value <0.05) (Fig. 6^19^). While each of the other 4 algorithms had several key genes that were not significantly correlated with OS (Logrank P-value ≥ 0.05). Where, the genes that are not significantly correlated to OS in each algorithm are as follows: K-means algorithm has one: SMC3; WGCNA algorithm has one: RBBP7; T-test has four: PPOX, LRRC14, PRCC, TBCE; DESeq2 algorithm has four: ADAMTS13, ANGPTL6, ECM1, CSRNP1.

**Figure 7:**
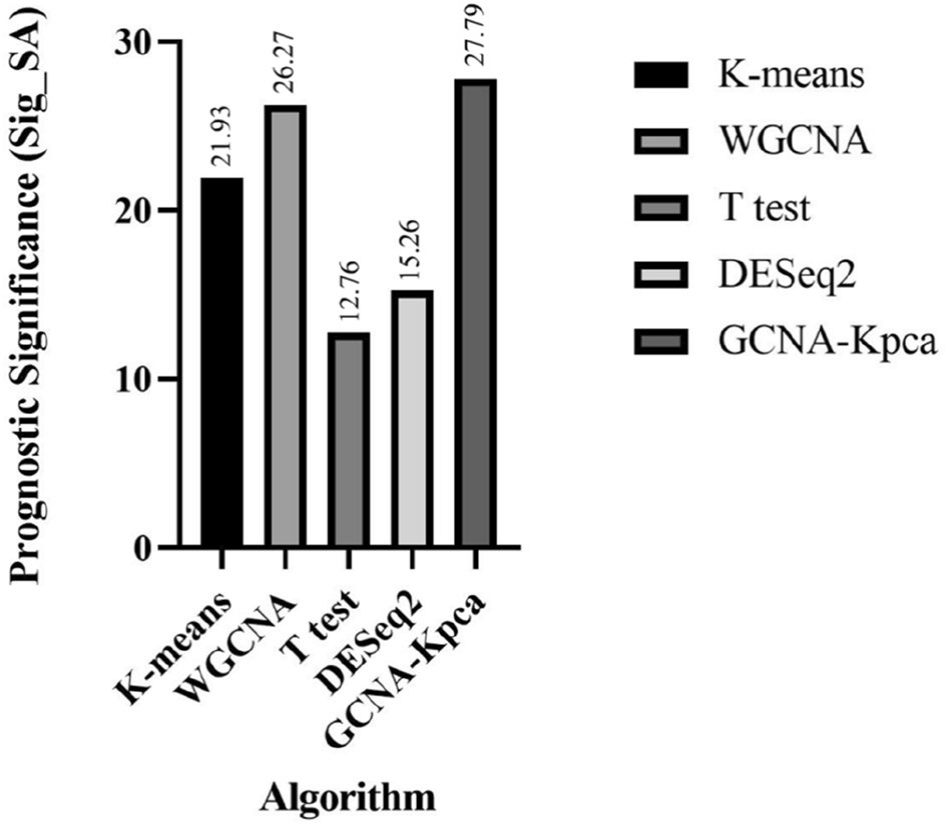
The prognostic significance of key genes obtained by K-means, WGCNA, T test, DESeq2 and GCNA-Kpca. This figure was drawn with GraphPad Prism 9.

Furthermore, formula (5) was used to calculate the prognostic significance of key genes obtained by each algorithm (Fig. 7). The results showed that the algorithm proposed in this paper had the highest prognostic significance (Sig_SA = 27.79).

### Verification of key genes identified by GCNA-Kpca algorithm

**Figure 8:**
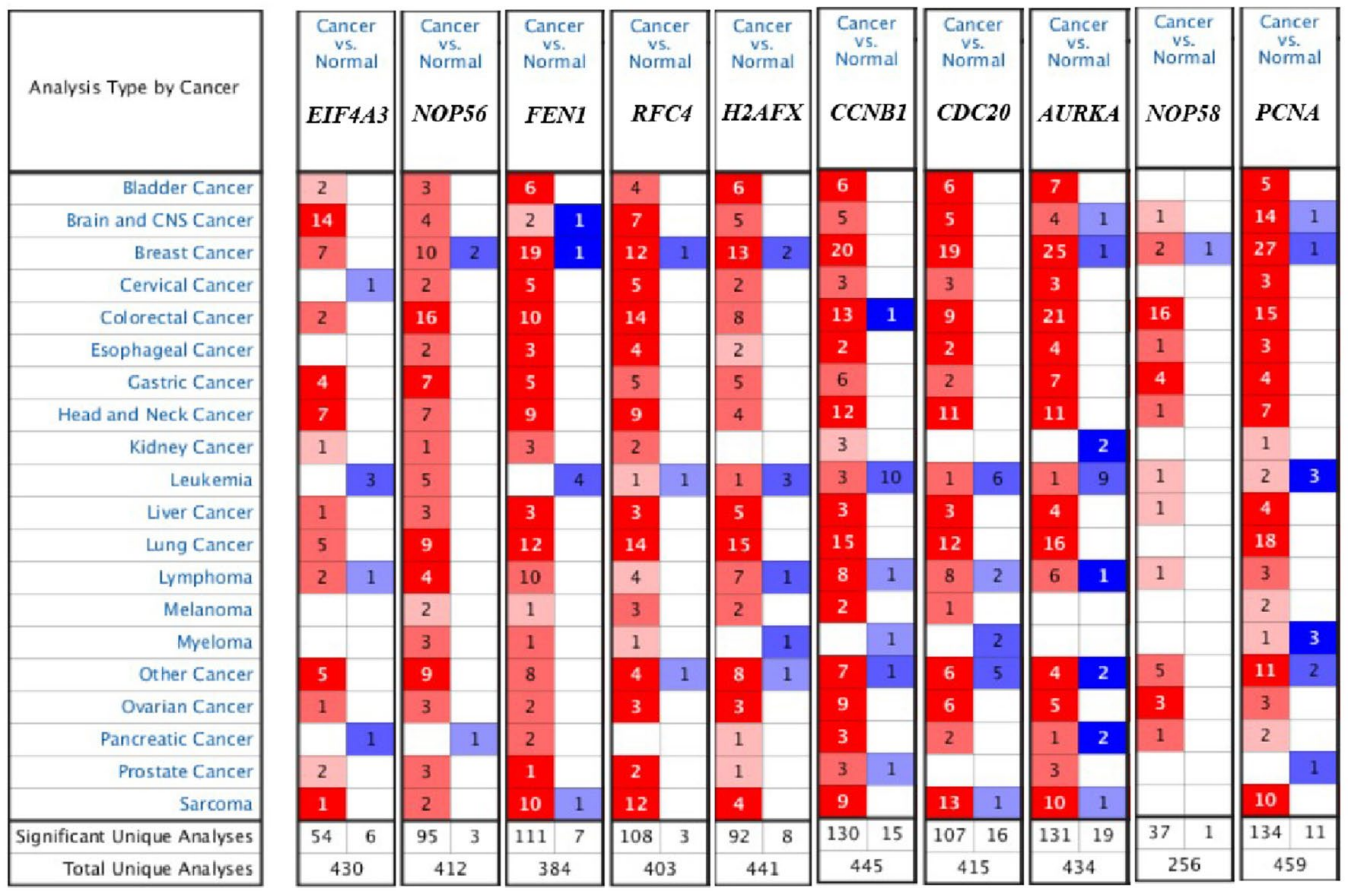
The results returned from Oncomine database. The row corresponds to cancer, and the column corresponds to gene. The red square represents that the gene was up-regulated in cancer, the blue square represents that the gene was down-regulated in cancer, and the value in the square represents the number of related references. This figure was drawn with Oncomine^15^ (https://www.oncomine.org).

**Figure 9:**
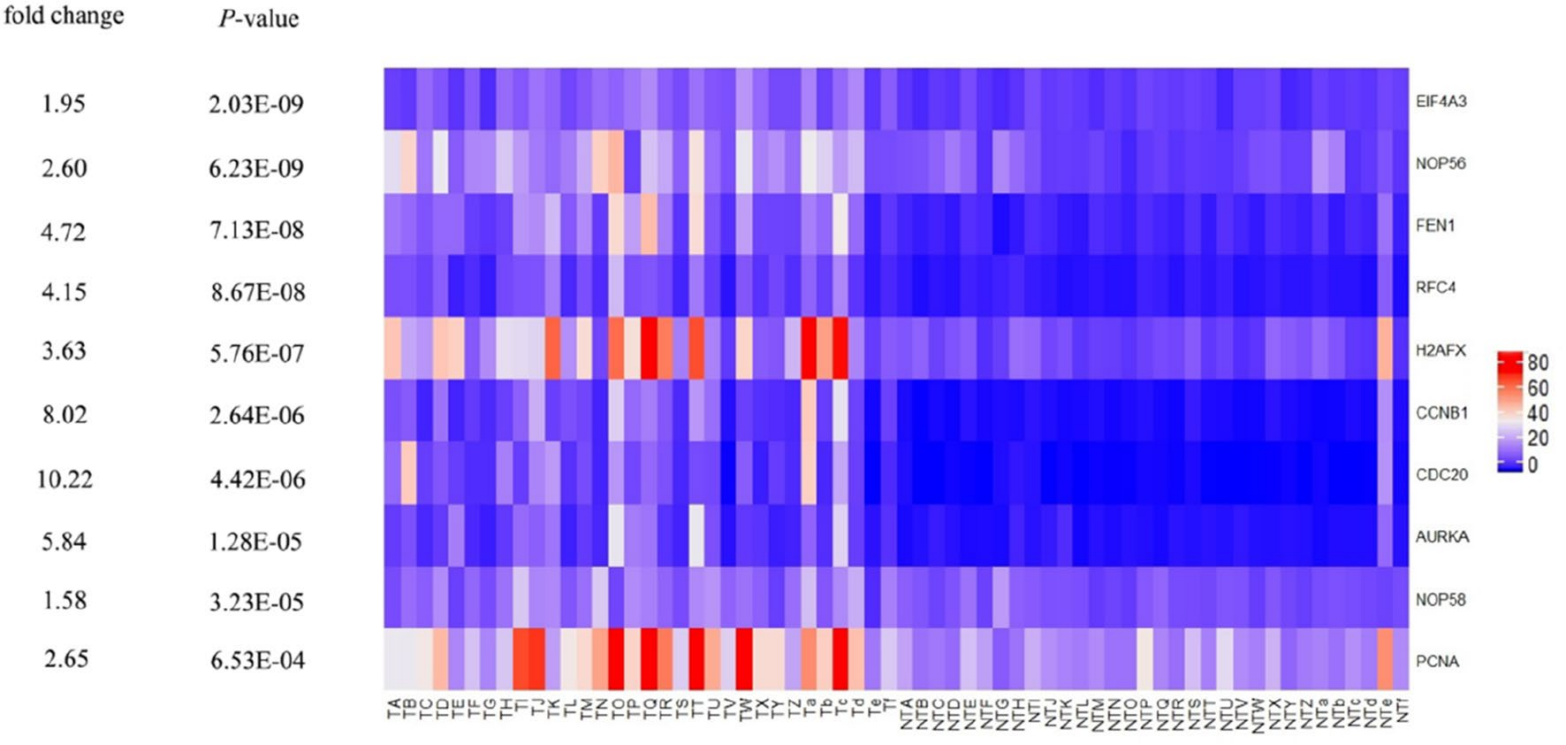
The heat map of RPKM of key genes identified by GCNA-Kpca algorithm in normal and HCC samples. TA-Tf represents HCC samples in GSE138485, NTA-NTf represents normal samples in GSE138485. This figure was drawn with R software^16^.

**Figure 10:**
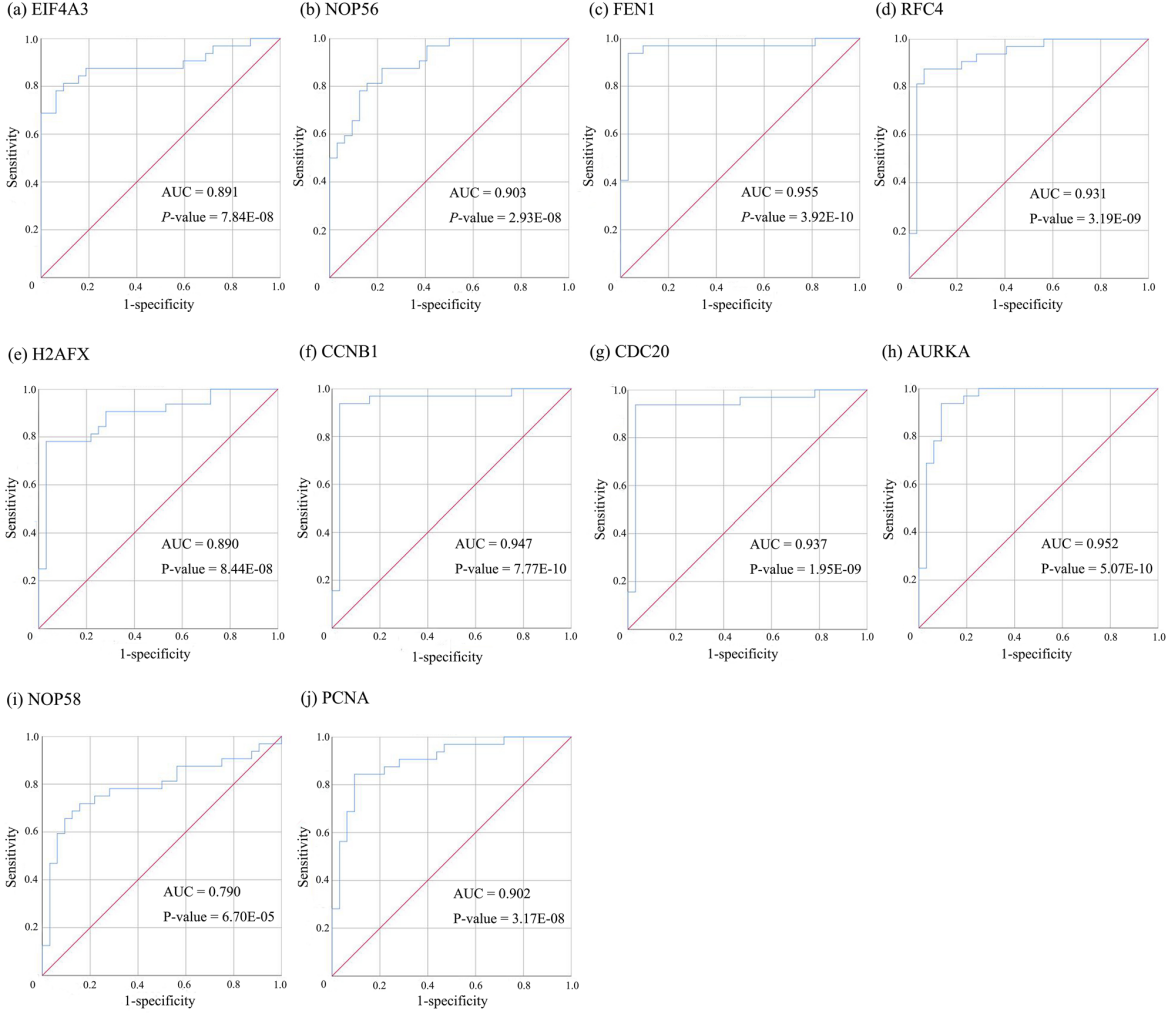
The ROC curves of key genes identified by GCNA-kpca algorithm. These ROC curves described the diagnostic efficiency of the mRNA levels of 10 key genes for HCC and normal tissues. This figure was drawn with IBM SPSS Statistics^25^.

We used three methods to further verify the role of key genes identified by GCNA-kpca algorithm: Firstly, the mRNA expression of 10 key genes in liver cancer was explored using Oncomine analysis. The result showed that all key genes were up-regulated in liver cancer as shown in Fig. 8. Then, the data of GEO (GSE138485) showed that the RPKM of these key genes were significantly (all P-values < 0.001) up-regulated in HCC samples compared with normal samples (Fig. 9). Moreover, based on the RPKM of these key genes in the GEO data set, we used ROC curve and AUC to classify HCC and normal samples. The results showed that the whole 10 key genes had highly diagnostic efficiencies to distinguish tumors from normal tissues (AUC > 0.79 and P-value < 0.0001) (Fig. 10).

## Discussions

HCC is the main type of liver cancer, and it causes the death of more than 700,000 patients every year. HCC is the third leading cause of cancer-related deaths in the world and has become an important issue affecting human health^20,21^. Previous studies focused on the specific genes in the initiation and progression of HCC^22–24^. Although some bioinformatics research on HCC has been reported^9,25^, but the precise molecular mechanisms underlying HCC progression was not clear. Therefore, the GCNA-Kpca algorithm was used to analyze the gene expression profiles of HCC and more accurately identify the gene modules and key genes in HCC, so as to further understand the pathogenesis of HCC.

GO enrichment analysis showed that the key gene module of HCC which obtained by GCNA-Kpca algorithm was related to many BPs. The top 20 GO terms with the lowest P value of BPs were divided into four categories with QucikGO (https://www.ebi.ac.uk/QuickGO/). Where, cell cycle phase transition (GO:0044770), mitotic cell cycle phase transition (GO:0044772), regulation of cell cycle process (GO:0010564), regulation of mitotic cell cycle (GO:0007346), cell division (GO:0051301), nuclear division (GO:0000280) and mitotic nuclear division (GO:0140014) are parts of cell cycle process (GO:0007049). Previous studies were shown that G2/M phase, apoptosis and cytoprotective autophagy was the key way to treat HCC^26^. Yan H et al. found that aberrant expression of cell cycle related genes (e.g., CDK1, CCNA2, CCNB1, BUB1, MAD2L1 and CDC20) and material metabolism related genes (e.g., CYP2B6, ACAA1, BHMT and ALDH2) may contribute to HCC occurrence^27^. Related studies had shown that Germline aberrations in critical DNA-repair and DNA damage-response genes caused cancer predisposition, whereas various tumors harbor somatic mutations causing defective DDR/DNA repair^28^. Moreover, aberrant activation of DNA repair was frequently associated with tumor progression and response to therapy in HCC^29^. And Lin et al. defined DNA repair based molecular classification that could predict the prognosis of patients with HCC^29^. Spindle organization (GO:0007051), mitotic spindle organization (GO:0007052) and microtubule cytoskeleton organization involved in mitosis (GO:1902850) belong to cytoskeleton organization (GO:0007010). Interestingly, Cheng et al. performed laser confocal technology and Immunohistochemical staining technique, and found that nuclear pleomorphism of cancer cells was correlated with the cytoplasmic disorganization of cytoskeleton^30^. RNA localization (GO:0006403) belongs to localization (GO:0051179). Cheng et al. found that differentially expressed cancer lncRNAs and lncRNAs with multiple cancer target proteins tended to have higher target location diversity in multiple cancers^31^. It could be seen that the BPs enriched by key module (obtained by GCNA-Kpca algorithm) were significantly correlated with the initiation and progression of cancer, which further proved that GCNA-Kpca algorithm had a good performance in gene module identification.

According to the validation, the 10 key genes obtained by GCNA-Kpca might be good biomarkers in HCC. The eukaryotic translation initiation factor 4A-3 (EIF4A3) is the core component of the exon junction complex (EJC). Based on the analysis of HCC sequencing data, researchers revealed the key role of EIF4A3 as a bridging protein, and believed that the abnormalities in EIF4A3 were related to carcinogenesis^32^. The flap structure-specific endonuclease 1 (FEN1) is over-expressed in a variety of malignant tumors, which may promote the invasiveness of tumor^33^. The expression levels of FEN1 were also positively correlated with tumor size (P = 0.047 < 0.05), distant metastasis (P = 0.013 < 0.05) and vascular invasion (P = 0.024 < 0.05) in HCC^34^. Human replication factor C4 (RFC4) is involved in DNA replication as a clamp loading agent and plays a role in a variety of cancers^35^. Studies had shown that the over-expression of RFC4 in tumor tissues was related to the poor prognosis of HCC, and it could be potential therapeutic targets for HCC^36^. In addition, RFC4 could enhance the repair effect of chemotherapeutic drugs on DNA damage^37^. H2A histone family, member X (H2AFX) is important in maintaining chromatin structure and genetic stability. Mutations in H2AFX may alter protein function, thereby altering cancer risk^38^. H2AFX were assessed by immunohistochemistry and/or immunoblotting and qRT-PCR in a collection of human HCC, and it was found that H2AFX was up-regulated in HCC^39^. Cyclin B1 (CCNB1) belongs to a highly conserved cyclin family, which is significantly over-expressed in many cancers^40^. Correlated with advanced histologic grade and/or vascular invasion, up-regulation of CCNB1 in HCC tissues predicted worse OS and disease-free survival (DFS) in HCC patients^41^. Cell division cycle 20 (CDC20) plays an important role in chromosome separation and mitosis^42^. CDC20 encodes a regulatory protein interacting with the anaphase-promoting complex/cyclosome in the cell cycle and plays important roles in tumorigenesis and progression of multiple tumors^43^. Immunohistochemistry result showed that, in the 132 matched HCC tissues, high expression levels of CDC20 were detected in 68.18% HCC samples, and over-expression of CDC20 was positively correlated with gender (P=0.013), tumor differentiation (P = 0.000), TNM stage (P = 0.012), P53 and Ki-67 expression (P = 0.023 and P=0.007, respectively)^44^. Aurora kinase A (AURKA) is an important regulator in mitotic progression and is often over-expressed in human cancers (including HCC)^45^. In fact, elevated AURKA expression was observed in several human cansers, such as pancreatic cancer, endometrioid ovarian carcinoma and colorectal cancer liver metastasis, and was associated with poor prognosis^46^. Moreover, AURKA regulated epithelial-mesenchymal transition and cancer stem cell properties in HCC to promote cancer metastasis^47^. Proliferating cell nuclear antigen (PCNA) plays critical roles in many aspects of DNA replication and replication-associated processes, including translesion synthesis, error-free damage bypass, break-induced replication, mismatch repair, and chromatin assembly^48^. Zheng et al. analyzed HCC data sets in GEO and TCGA and found that PCNA might be promising prognostic biomarker for HCC^49^. Nucleolar KKE/D repeat proteins NOP56p and NOP58p interact with NOP1p and are required for ribosome biogenesis^50^. Strikingly, NOP56p and NOP58p are highly homologous (45% identity). NOP56 is a nucleolar protein that closely relates to the expression oncogene^51^. Interestingly, NOP56 and NOP58, all from the key gene module, have not been shown to be associated with HCC to date, either in vivo or in vitro. But studies had shown that FAM83A-AS1 facilitated HCC progression by binding with NOP58 to enhance the stability of FAM83A^52^. Combined with the study in this paper, it was reasonable to speculate that these 10 key genes could be biomarkers for HCC. It is worth noting that NOP56 and NOP58 are the HUB genes of HCC that we discovered for the first time. But the key role of these two genes still needs to be verified by subsequent biological experiments. And it further proved the good performance of GCNA-Kpca algorithm in key gene identification.

WGCNA is the most classic method in gene module identification. However, WGCNA algorithm didn’t take modularity into account in gene module identification, and it could not find the best membership module for each gene through multiple iterations, so that its module identification effect was not ideal. To solve this problem, a gene module identification algorithm based on Newman algorithm and K-means algorithm framework, GCNA-Kpca algorithm, was proposed. The results showed that compared to the other seven clustering algorithm, the GCNA-Kpca algorithm had the best performance in error rate, biological significance and CNN classification indicators (*Precision*, *Recall* and *F-score*). Moreover, the key gene identification results showed that all key genes identified by the GCNA-Kpca algorithm could be used as prognostic targets; And compared with the other four algorithms, the key genes obtained by this algorithm had the highest prognostic significance. It not only proved the reliability of the gene modules identified by the GCNA-Kpca algorithm, but also suggested that this algorithm could play a good performance in the identification of biomarkers and prognostic targets.

## Conclusions

Taken together, GCNA-Kpca, a gene module identification algorithm combined with Newman algorithm and K-means algorithm, was proposed in this paper, and the gene expression profiles of HCC were analyzed by this algorithm. The results showed that the gene modules identified by this algorithm had the highest biological significance. Moreover, all key genes identified by the GCNA-Kpca algorithm could be used as prognostic targets, and these key genes had the highest prognostic significance. Notably, NOP56 and NOP58 are key genes for HCC that we discovered for the first time. The experimental results showed that this algorithm performed well in the identification of gene modules and key genes.

## Supplementary Information

Supplementary Figure S1.

Supplementary Table S1.
