# Supplementary figures and images for: A gene module identification algorithm and its applications to identify gene modules and key genes of hepatocellular carcinoma
Source: Sci Rep. 2021 Mar 9;11:5517. doi: 10.1038/s41598-021-84837-y (PMC7943822; doi:10.1038/s41598-021-84837-y)

**Figure S1 Hierarchical clustering tree of HCC samples.**

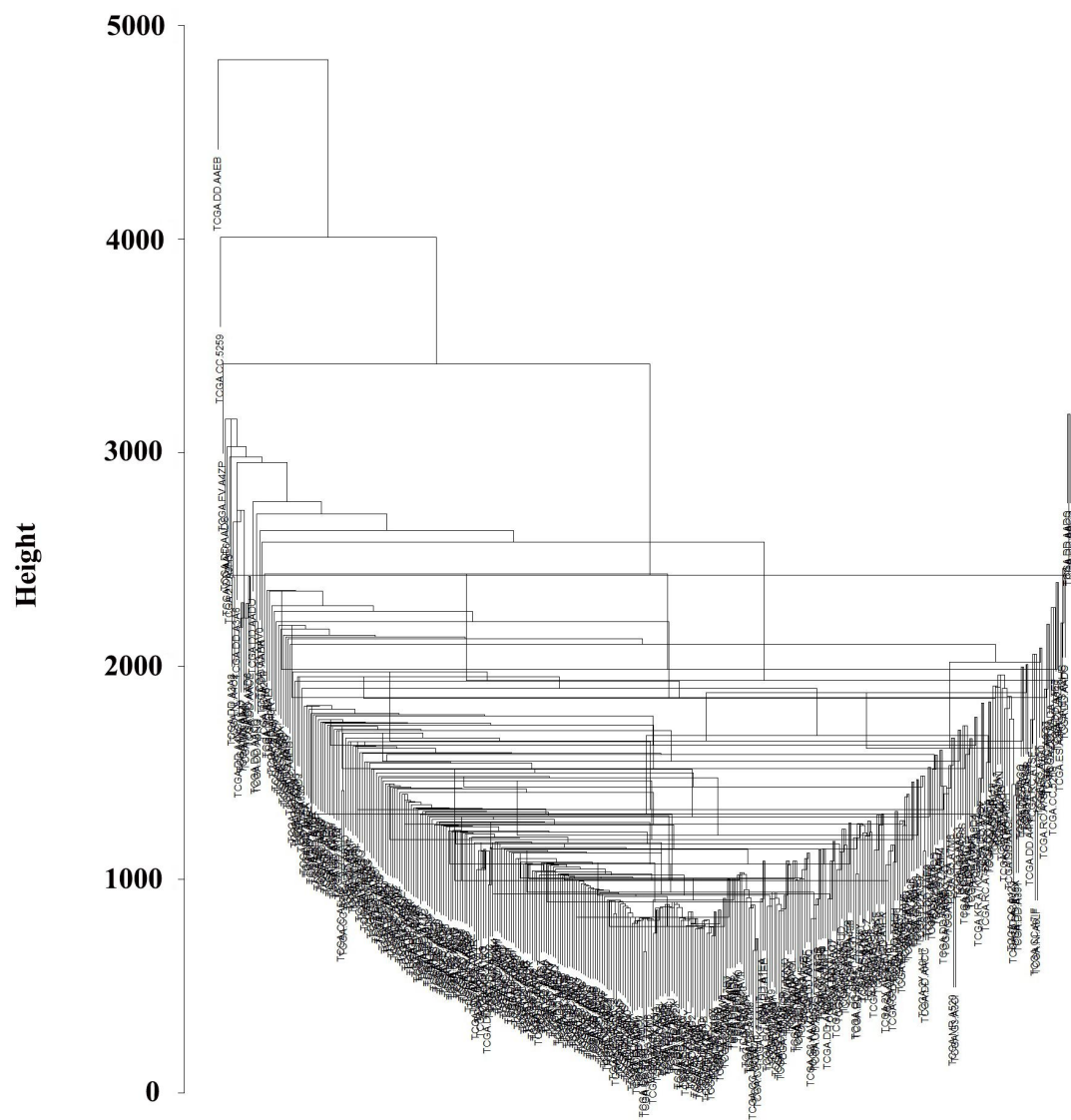

Supplement: Supplementary file 1 — Supplementary Figure S1. [file 41598_2021_84837_MOESM1_ESM.pdf]
